# A systematic review and meta-analysis of the prevalence and predictors of anemia among children in Ethiopia

**DOI:** 10.4314/ahs.v20i4.59

**Published:** 2020-12

**Authors:** Alemu Gebrie, Animut Alebel

**Affiliations:** 1 Department of Biomedical Science, School of Medicine, Debre Markos University, Debre Markos, Ethiopia; 2 Department of Nursing, College of Health Sciences, Debre Markos University, Debre Markos, Ethiopia

**Keywords:** Anemia, prevalence, predictors, children, Ethiopia, systematic review, meta-analysis

## Abstract

**Background:**

Anemia is a wide-spread public health problem characterized by a decrease in hemoglobin concentration and/ or red blood cell volume below an established cut-off value. In developing countries including Ethiopia, about half of children are estimated to be anemic. Therefore, the purpose of this study was to determine the pooled prevalence of anemia and its predictor factors among children in Ethiopia.

**Method:**

The studies were identified through explicit and exhaustive search of reputable databases (PubMed, Google scholar, Science Direct, EMBASE, Cochrane library, and the hand search of reference lists of previous prevalence studies to retrieve more related articles. Thirty-nine studies were selected based on a comprehensive list of inclusion and exclusion criteria. Data were extracted using a standardized and pre-tested data extraction checklist, and the analysis was done using STATA 14 statistical software. To assess heterogeneity, the Cochrane Q test statistic and I2 tests were used. In our analysis, considerable heterogeneity was observed. Therefore, a random effect meta-analysis model was used to estimate the pooled prevalence of anemia. Moreover, the predictor factors of anemia were examined.

**Results:**

The forest plot of 39 included studies revealed that the overall pooled prevalence of anemia among children in Ethiopia was 34.4% (95% CI: 29.1, 39.7%). Sub-group analysis showed that the highest anemia prevalence was observed in Somali Region with a prevalence of 49.4 % (95% CI: 20.9, 77.8). Also, anemia in children was found to be highest in the age group of less than five years (45.2, 95% CI: 39.6,50.8). Low literacy of families: 1.3 (95% CI: 1.1, 1.7), low family socioeconomic status: 1.9 (95% CI: 1.1,3.01.3), having housewife mothers or with no job: 1.5 (95% CI: 1.4, 1.9) and rural residence: 3.3 (95% CI: 1.7,6.1) were found to be predictors of anemia among children.

**Conclusion:**

In this study, one in three children were anemic in Ethiopia. It is a moderate public health problem in children in this study. Low literacy, low socioeconomic status as well as rural residence of the families and helminthic infection of the children were found to be predictors of anemia in the children. Community and school-based interventions should be strengthened to improve the problem.

## Background

Anemia is a wide-spread public health problem characterized by a decrease in hemoglobin concentration and/or red blood cell volume below an established cut-off value resulting in an impaired capacity of the blood to transport oxygen to the body tissues 1, 2. Worldwide, it is estimated that more than 2 billion people are suffering from anemia. Although it occurs at all population, anemia is more pervasive in pregnant women and school aged children 3, 4. In developing countries, about half of children are estimated to be anemic, and it is incommodious in sub-Saharan African Countries like Kenya 48.9%^5^, Mali 55.8% 6 and Tanzania 79.6%^7^. As per the 2016 report of Ethiopian Demographic and Health Survey (EDHS), more than half (57%) of Ethiopian children aged 6–59 months are anemic. The figure is higher than that reported in EDHS 2011 (44%), which is above the cut off value (40%) of the World Health Organization (WHO) category of anemia as a severe public health problem 8, 9. The EDHS 2016 report also showed that mild, moderate and severe anemia accounted for 25%, 29% and 3%, respectively, and children in rural areas are at a higher risk to be anemic than children in urban areas (58% vs 49%) 10.

Anemia is the result of multiple risk factors that vary across geographical areas, and coexist together with deficiencies of other micronutrients, of which iron deficiency is the leading (50%) predictor^11^, ^12^. Studies unveiled that socioeconomic factors like illiteracy^13^, gender norms 14, and poverty 15; infectious diseases such as intestinal helminths, tuberculosis and malaria 7, 16 are risk factors for anemia. In addition, genetic problems, digestive abnormalities, absorption difficulties as well as the deficiencies of other essential micronutrients including folic acid, vitamin B12 and vitamin A contribute to the occurrence of anemia 17.

Anemia results in several acute and chronic health problems on children. It impairs their learning performance, psychomotor and cognitive maturity, behavioral and physical growth increasing the risk of morbidity and mortality 2, 18. In addition, anemia is related to poor intelligent quotient, and it also affects the capacity of language coordination in children. Generally, anemia is one of the public health challenges for the country and it is an indicator of poor health status 3, 4.

Integrated into the ordinary childhood health services and community based nutritional interventions, the Ethiopian Ministry of Health has been implementing anemia prevention programs to control childhood anemia. Moreover, the ministry has regularly performed children de-worming movements since 2004 19. Despite efforts to combat anemia in children in the last two decades, it remains a major health problem of children in the country 9. Because of the presence of complex and multifactorial risk factors for anemia and the potential interactions among them, the implementation of highly integrated strategy for prevention and control of anemia in children is imperative 20.

In different parts of Ethiopia, several independent and fragmented studies as well as a review study 9, 21–59 were carried out in children to assess the prevalence and associated factors of anemia, but there was a great variation and inconsistency among the findings of the studies. Also, the previous review study did not include relevant studies exhaustively, and did not review more important predictors of anemia among children. In addition, important statistical analyses were missing in the review. Hence, the aim of this systematic review and meta-analysis was to review and determine the pooled prevalence and predictor factors of anemia among children in Ethiopia. The findings from the present study will help for policy makers, program planners, guardians or parents, clinicians as well as concerned stakeholders to give more emphasis for childhood anemia in the country. The study will also be of paramount importance for contemporary researchers to be contentedly engaged in related topics. The systematic review question is: What is the best available evidence on the prevalence and predictors of anemia among children in Ethiopia?

## Methods

### Study design and literature searching strategy

We followed the methods of Gebrie et al., 2018 60. We carried out a systematic review and meta-analysis of eligible published and unpublished studies to determine the pooled prevalence of anemia and its predictors among children in Ethiopia. The Preferred Reporting Items for Systematic Reviews and Meta-Analyses (PRISMA)^61^ guideline was followed for the scientific rigor of the study. The studies for this review were retrieved through reproducible and comprehensive electronic searching of major reputable databases (PubMed, Google scholar, ScienceDirect, EMBASE, Cochrane library). We have also done manual searching of the reference lists of already identified relevant articles so as to retrieve more eligible studies. In addition, we used the “related articles” option of PubMed to search more relevant articles. Moreover, an open search in national and Ethiopian Ministry of Health websites were done to identify anemia prevalence among children not reported in scientific journals. The two authors (AG, AA) performed the search independently based on the following key terms: (1) population (preschool, children, schoolchildren, school aged, childhood, schooler, preadolescent); (2) outcome (anemia, anaemia, hematologic parameter, hematologic profile, micronutrient deficiency; (3) study design (prevalence, cross-sectional, epidemiology, observational, longitudinal study); and (4) location (regions of Ethiopia and Ethiopia). The terms were used both separately and in combination with the help of the Boolean operator like “AND”, “OR”, or “NOT” (see additional file 1). We have meticulously analyzed the appropriateness of the searching terms before performing the search in order to identify the relevant studies. The literature search was limited to human study category and English language. However, the literature search for published articles was not restricted by time, and all the studies up to January 03/2018 were considered to be included in this systematic review and meta-analysis. The articles were searched, organized and extracted from September, 2017 to January 03/2018. Also, End-Note X7 reference manager has been used to manage the literature retrievals since the inception of the study.

### Study selection

#### Inclusion criteria

The two authors independently and meticulously appraised the contents of each of the identified articles (AG and AA). The studies which met the following criteria were considered eligible for inclusion in the study. The retrievals were appraised for inclusion in the final review by using their titles, abstracts, and then by reviewing full text papers.

**Population:** Studies carried out in children

**Study area:** Those studies conducted only in Ethiopia.

**Study design:** Original studies and government surveys which reported the prevalence and predictor factors of anemia among children in Ethiopia were included. Language: only studies reported in English language were included.

**Publication condition:** Studies which meet the eligibility criteria were included regardless of their publication status (published, unpublished and grey literature, etc.)

#### Exclusion criteria

The two reviewers (AG and AA) performed the selection of the studies independently and blindly after thorough screening of the abstracts and the full texts of the studies. Those articles having methodological problems were excluded. Any disagreements during review process were resolved by consensus and when the disagreements continue after discussion, a third person was consulted to finally settle the discrepancy. The processes of identifying, screening and including or excluding records were done using the PRISMA 61 flow diagram guideline.

### Data abstraction and quality assessment

The two authors abstracted the required data using a pilot-tested and prepared data abstraction tool. The extracted data included: first author, area where the study was conducted, study design, publication year, sample size, response rate, mean hemoglobin level, and prevalence of anemia among children. Any sort of disagreement during the data extraction were resolved by discussion as well as through involvement of the third person (AZ).

The authors employed the Newcastle-Ottawa quality assessment tool Scale adapted for cross-sectional studies in order to assess the qualities of the studies 62. This standardized tool contains three main indicators regarding the methodological qualities, the comparability and the statistical analyses of articles. The qualities of the studies were evaluated by using the following indicators; those with medium (fulfilling 50 % of quality assessment criteria) or high quality (≥6 out of 10 scales) were included in the study. The assessment results were determined taking the mean score of the two authors.

### Outcomes of interest of the study

The foremost outcome of the current study was prevalence of anemia among children. According to WHO 2001 63, anemia among children was defined as a hemoglobin concentration of less than 11 g/dL for ages less than 5 years, hemoglobin concentration of less than 11.5 g/dL for ages 5–11 years, and hemoglobin concentration of less than 12 g/dL for ages 12–14 years. As per WHO 2011 guideline 64, anemia was also defined as hemoglobin concentration less than 11 g/dl for children less than 5 years old, hemoglobin concentration less than 11.5 g/dl for children 5–11.9 years old, and hemoglobin concentration less than 12 g/dl for children 12–14.9 years old. The studies included in this review operationalized and defined anemia as per WHO guidelines, some of them adjusting for altitude. The second outcome of this study was associated factors of anemia among the study subjects. The prevalence was obtained by dividing the number of children who are anemic to the total number of children included in the study (sample size) then multiplied by 100. The association between anemia and the predictors were quantified by odds ratio. The odds ratio was calculated from the two by two table reports of the original studies.

### Heterogeneity and publication bias

Publication bias and heterogeneity were checked using the Egger's and Begg's tests. A p-value of less than 0.05 were considered to declare statistical significance of publication bias and heterogeneity. The heterogeneity of studies was also assessed using I2 test statistics. The I2 test statistics of 25%, 50%, and 75% was considered as low, moderate and considerable heterogeneity, respectively. For the test results exhibited heterogeneity, random effect model was used as a method of analysis.

### Data analysis/synthesis of results

After the relevant data had been extracted from the studies by using Microsoft Excel 2016 format, the authors then analyzed the results by using STATA version 14.0 (STATA Corporation, College Station Texas) software. The original studies were summarized and presented by using a table and a forest plot. The authors computed the standard error of prevalence of anemia for each original study by using binomial distribution formula. We explored the potential heterogeneity among the reported prevalence of the studies using I2 test and Cochrane Q statistics 65/a>. Since the test statistics revealed that there was a considerable heterogeneity^66^ among the studies (I2 = 99.5%, p <0.001), a random effects model was used to estimate the Der Simonian and Laird's pooled effect. We have also undertaken univariate meta-regression analysis taking publication year of the studies and the sample size to detect the potential source(s) of variation but both of them were found to be statistically insignificant, (p>0.05 and p>0.05 respectively). Potential publication bias was also objectively examined using Egger's weighted correlation and Begg's regression intercept tests at 5% significant level respectively 67, 68. The test results showed that there is no significant publication bias (p> 0.05). the funnel plot also supports subjectively the absence of publication bias (see additional file 2). In addition, to minimalize the random variations between the point estimates of the original studies, subgroup analysis was carried out based on region of studies, study settings and age group of children.

## Results

### Search results

**Figure 1:**
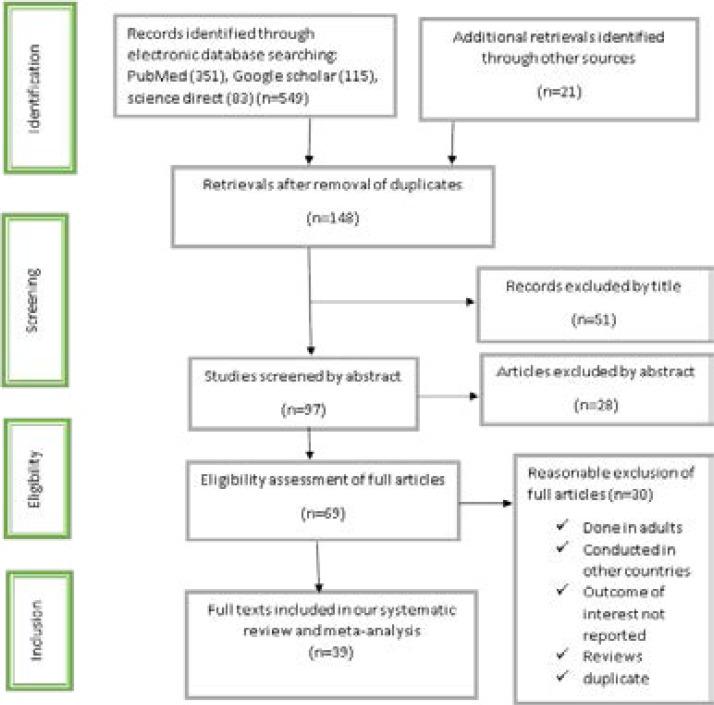
Flow chart diagram and PRISMA checklist describing selection of studies for the systematic review and meta-analysis of prevalence and predictors of anemia among children in Ethiopia, 2018 (identified, screened, eligible and included studies). Articles may have been excluded for more than one reason.

From the beginning, we retrieved a total of 570 records through manual and electronic searches. The electronic search was performed through database search of MEDLINE/PubMed, Google scholar, science direct, EMBASE, Cochrane Library and reference lists of previous related studies to retrieve more related articles. Because of duplications in the records, 422 of them were removed from the retrievals. After the authors assessed the titles and abstracts of the records, the remaining 148 retrievals, 79 records were excluded for they were not relevant for this review in terms of outcome the study is interested. Then, 69 full text were considered and assessed for eligibility based on the preset eligibility criteria. Finally, 39 studies were considered to be relevant and included in this systematic review and quantitative meta-analysis (Figure 1).

From a total of 69 full text studies accessed, we removed nine of them because the outcome of interest was not mentioned and/or they were conducted in other nations which are not the location of interest of the review; these studies were conducted in Africa 69, 70, Tanzania^71^, ^72^, Malawi 73, Nigeria 74–76 and Mali 77. In addition, 20 full text articles 78–97 that have been carried out from different parts of Ethiopia were excluded because their outcome measures were not prevalence of anemia in children, and they were conducted in the adult population which is not the population of interest of the this meta-analysis. Moreover, one full text article 98 was excluded because of duplicated publications of our result of interest in different journals (see additional file 3).

### Description of original studies

Table 1 presents the descriptive summary of the characteristics of 39 studies included in this systematic review and meta-analysis. All of the studies are cross sectional by design (community based, school based and health institution based, see additional file 4). The studies were conducted in different regions of Ethiopia with a sample size that ranges from 64 in JUSH (Jimma University Specialized Hospital), Oromia region to 9267 in EDHS 2016. The eligible studies have been carried out since 1999. In the present systematic review and meta-analysis, a total of 64,727 children were enrolled to estimate the pooled prevalence of anemia in the subjects.

**Table 1:** Characteristics of 39 studies reporting the prevalence of anemia among children in Ethiopia included in the current systematic review and meta-analysis, 2018 Hb* = hemoglobin, N/A= Not Applicable

Region	Area	Author	Publication year	Sample size	Response rate (%)	Quality score (10 pts)	Mean Hb* (in g/dl)	Prevalence of anemia
**Addis Ababa**	Yekatit 12 hospital	Debasu et al 41	2015	106	100	6	12.2	18.9
	Zewditu hospital	Mihiretie et al 36	2015	180	N/R	7	-	22.2
**Afar**	Amibara woreda	Deribew et al 55	2013	387	100	7	9.67	31.8
**Amhara**	Libo Kemkem and Fogera	Herrador et al 52	2014	764	85.9	8	-	30.9
	Durbete	Alelign et al 23	2015	384	95.3	7	-	10.7
	Wag-Himra	Woldie et al 47	2015	347	97	7	-	66.6
	Gondar	Enawgaw et al 34	2015	264	99.6	8	-	16.2
	Amhara	Gashu et al 35	2016	628	628	6	-	13.6
	Gondar university hospital	Geletaw et al 33	2017	222	91.7	6	11.5	42.8
	Gondar town	Getaneh et al 45	2017	523	96.5	8	-	15.5
	BDR and Mecha district	Feleke et al 42	2017	2372	94.5	6	11.6	7.6
	Debre birhan	Engidaye et al 58	2017	432	100	7	-	28.5
**Benishangul**	Pawe General Hospital	Birhanu et al 32	2017	377	100	6	11.7	40.3
**Harari**	Babile	Teji et al 22	2017	547	91	5	-	32
	Hiwot Fana hospital	Teklemariam et al 38	2015	103	N/R	7	10.7	54.4
**Nation wide**	Ethiopia	Habte et al 29	2013	8260	100	6	10.7	50.3
	Ethiopia	Reithinger et al 30	2013	6054	92	7	-	36.4
	malaria endemic arias	Birhanu et al 21	2017	763	73.1	8	12.8	17.3
	Nation wide	EDHS 57	2005	4138	N/R	N/A	-	53.5
	Nation wide	EDHS 56	2011	9157	93.4	N/A	-	44.2
	Nation wide	EDHS 9	2016	9267	88	N/A	-	56.9
**Oromia**	JUSH	Gedefaw et al 31	2013	234	100	7	14.1	23.1
	JUSH	Muluneh et al 51	2009	64	94.1	5	10.5	21.9
	Jimma Town	Assefa et al 25	2014	404	95.6	7	11.6	37.6
	Jimma Town	Desalegn et al 26	2014	586	95	8	-	43.7
	Kersa district	Mesfin et al 24	2015	1755	N/R	8	12.6	27.1
	Butajira	Taye et al 37	2015	739	73.4	7	11.8	34.8
	Jimma Health Center	Seble et al 53	2016	130	51.8	8	-	33.1
	Adami Tullu	Gari et al 40	2017	6112	N/R	8	11.6	32.6
**Oromia & Tigray**	Babile and Enderta	Roba et al 46	2016	216	98.2	7	11.4	53.7
**SNNP**	Gurage	Birmeka et al 48	2017	680	97.4	5	-	31.3
	Wolaita Zone	Tiku et al 44	2018	404	N/R	8	-	51.4
	30 schools	Grimes et al 43	2017	3729	99.4	7	-	23
**Somali**	Filtu	Gutema et al 28	2014	355	100	8	12.4	23.7
	Gode zone	Guled et al 49	2017	397	100	6	9.7	72
	Kebribeyah refugee	Jemal et al 50	2017	399	90.7	6	-	52.4
**Tigray**	Mekele, Quiha and Aynalem	Adish et al^54^	1999	2080	88	5	-	42
	Mekele	Mahmud et al^39^	2013	600	100	6	13.2	11
	Kilte Awulaelo	Gebremedhin et al 27	2014	568	100	7	11.5	37.3

The 39 studies have been done in almost all regions of Ethiopia: two of the studies were conducted in Addis Ababa; nine in Amhara region, one in Afar region, two in Harari region, one in Benishangul region, eight in Oromia region, one in Oromia and Tigray regions, three in Somali region, three in Tigray region, three from Southern Nations, Nationalities and peoples' region (SNNPR) and six nationwide survey studies three of which are EDHSs. The highest prevalence of anemia (72%) was observed in Somali Region (Gode Zone) in 2017. On the other hand, the lowest prevalence (7.6%) of anemia was reported in Amhara Region (Bahirdar and Mecha district schools) in the same year. Some of the studies also reported mean hemoglobin lebel. Besides, the primary studies included in this review had a response rate that ranges from 51.8% to 100%, and virtually all of the studies had good response rate (Table 1).

Regarding the publication status of the studies: only one 58 of the 39 studies is yet to be published. Also, three of the studies 9, 56, 57 are EDHS studies. However, the rest of the studies included in the current meta-analysis were retrieved by exhaustive and reproducible search from reputable databases such as PubMed. The authors meticulously appraised all the studies before review and analysis, and the included articles were confirmed fit for their quality i.e. quality score ranging from 5–8 out of 10 marks.

### Meta-analysis and meta-regression

**Figure 2:**
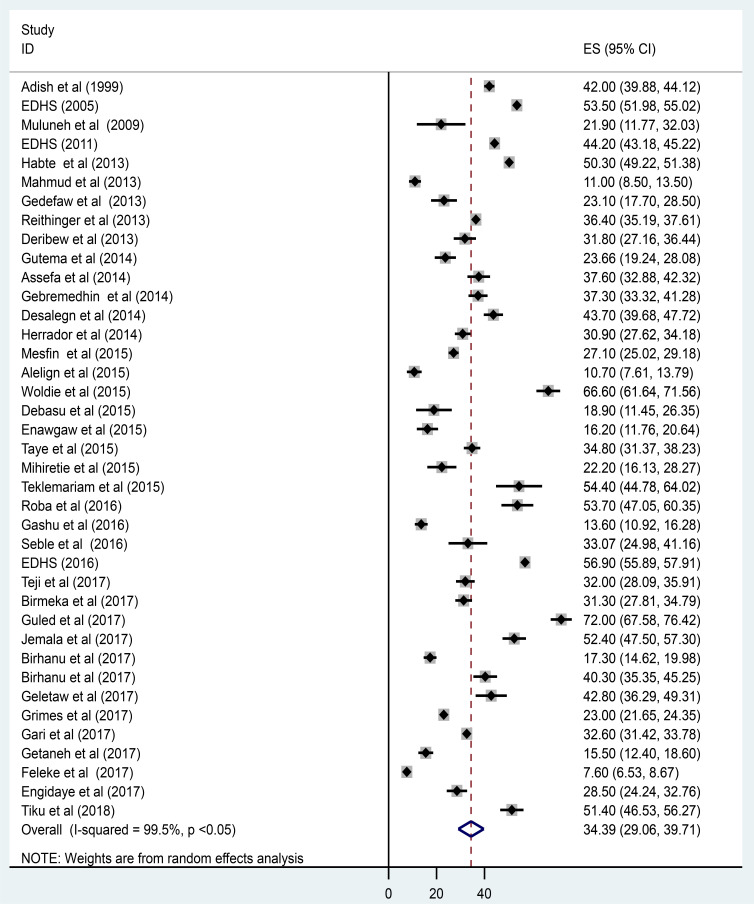
Forest plot of the pooled prevalence of anemia among children in Ethiopia, 2018

In this meta-analysis, a forest plot (Figure 2) was used to estimate the pooled effect size and the effect of each study with their respective confidence interval (CI) in order to provide a visual summary data. Herein, the forest plot of 39 included studies depicted that the overall pooled prevalence of anemia among children in Ethiopia was 34.4% (95% CI: 29.1, 39.7%). Detected by I2 statistic (I2 = 99.5, p value < 0.001), we observed considerable heterogeneity 66 across the included studies. As a result, we employed the DerSimonian and Laird random-effects model to estimate the overall pooled prevalence of anemia among children since the model gives more conservative effect size. Taking publication year and sample size of the studies as potential factors associated with the prevalence variation, we performed univariate meta-regression analysis to determine the likely sources for the variation. However, neither of them is statistically significant for the variation (Table 2). In addition, Egger's and Begg's tests revealed that there is no statistically significant publication bias, (p>0.05) and (p>0.05), respectively.

**Table 2:** Related factors with the heterogeneity of anemia prevalence among children in Ethiopia in the meta-analysis (univariate meta-regression), 2018

Variables	Coefficient	P-value
Publication year of studies	-1.4061	0.351
Sample sizes of the studies	0.0012186	0.603

### Sub-group analysis

Based on the region and the setting where the studies were conducted as well as the age group of children, we carried out subgroup analyses to assess the possible sources of heterogeneity. The result showed that the highest prevalence of anemia among children was observed in Somali Region with a prevalence of 49.4 % (95% CI: 20.9, 77.8) followed by nationwide studies, 43.2 (95% CI: 35.1, 51.2). But, the prevalence of anemia in the subjects was the least in Addis Ababa followed by Amhara region, 25.7 (95% CI: 15.9, 35.5), as compared to all other regions 21.4 (95% CI: 17.7, 25.1) (Table 3). Regarding the study settings, the prevalence of childhood anemia was relatively higher in studies which have been conducted in the community, 40.6 (95% CI: 35.4, 45.9) as compared to those studies carried out in health institution and school settings (Table 3). Also, anemia in children was found to be highest in the age group of less than five years (45.2, 95% CI: 39.6,50.8) (Table 3).

**Table 3:** Results from subgroup analysis of the prevalence of anemia among children in Ethiopia, 2018 (n = 39)

Variables	Characteristics	No studies	Prevalence with 95%	I^2^	p-value
**By region**	Addis Ababa	2	20.9 (16.2, 25.6)	0.0%	=0.501
	Amhara	9	25.7 (15.9, 35.5)	99.0%	<0.001
	Oromia	8	32.3 (28.4, 36.2)	90.9	<0.001
	Somali	3	49.4 (20.9, 77.8)	99.1	<0.001
	Others	11	36.8 (29.2, 44.4)	98.4	<0.001
	Nationwide	6	43.2 (35.1, 51.2)	99.6	<0.001
**By setting**	School based	9	34.1 (28.8, 39.3)	98.8%	<0.001
	Health institution	10	34.0 (22.5, 45.4)	97.1%	<0.001
	Community based	20	40.6 (35.4, 45.9)	99.3	<0.001
**By age group**	<Five years	14	45.2 (39.6,50.8)	99.2	<0.001
	>Greater than five	13	25.0 (18.8,31.2)	98.8	<0.001
	All age group	12	31.8 (19.6, 44.1)	99.2	<0.001

### Predictors of anemia among children

The authors did comprehensively review and meta-analyze the predictors of anemia among the study subjects using nineteen meta-analyzable studies 21, 23–28, 34, 36, 37,40–43, 45–47, 49, 50 from the relevant articles included in the current study. Sex of the children, family educational status, family occupation, family income, residence and helminthic infection were found to be worth reviewing and meta-analyzable. Except sex of the children, all the predictors revealed statistically significant association with anemia among children in Ethiopia (Table 4). The authors also performed sensitivity analysis for each of the factors, and none of the studies showed significant difference.

**Table 4:** Odds ratio of the associations between anemia and its purported associated predictor factors among children in Ethiopia, 2018

Variables	OR (95% CI)	I^2^	P-value
Sex	1.13 (0.99,1.28)	49.1%	0.019
Family education	1.34 (1.07,1.67)	67.9%	0.001
Family occupation	1.46 (1.14,1.88)	0.0%	0.608
Family income	1.86 (1.14,3.01)	92.8%	0.000
Residence	3.25 (1.72,6.13)	80.8%	0.000
Helminthic infection	2.52 (1.31,4.86)	93.7%	0.000

The pooled estimate of fourteen studies showed that male children were 1.1 times more likely to be anemic than their female counterparts, odds ratio 1.1 (95% CI: 1.0, 1.3) (Table 4). Those children whose families are illiterate (not educated) were 1.3 times more likely to be anemic as compared to those whose families are literate, odds ratio 1.3 (95% CI: 1.1, 1.7) (Table 4). In addition, those children whose mothers were housewives or with no job were 1.5 times more likely to be anemic as compared to those whose mothers had different types of jobs, odds ratio 1.5 (95% CI: 1.4, 1.9) (Table 4).8). Although there has been no similar study for comparison in the country, the 2016 Ethiopian demographic and health survey (EDHS) report revealed a higher (56.9%) prevalence of anemia among children in the country. Sampling and study period could be the possible reasons for the difference in the results. Even though the EDHS was conducted in a nationally representative sample of 9267, this meta-analysis included studies which have been conducted since 1999 with a total of 64,727 children.

Sub-group analyses results in this study revealed that the prevalence of childhood anemia substantially varied across regions of Ethiopia, in study settings and age group. The highest anemia prevalence among children was observed in Somali region (49.4 %) whereas anemia was less prevalent in Addis Ababa (20.9 %) and Amhara region (25.7 %). The variation in the prevalence of anemia among the regions in Ethiopia could partially be due to the difference in the economic, sociodemographic and dietary difference among the regions. Also, the difference in study numbers included in each category (region) might also be attributed for the variation. In addition, the prevalence of anemia was relatively higher in those studies conducted in the community settings than health institutions or schools. This could possibly be due to a low nutritional intervention coverage to reduce anemia prevalence. Finally, anemia in children was found to be higher in the age group of less than five years. This finding was in line with findings in other related studies 99–102. This could be because of the increased growth requirements in children under the age of five 102. This suggests that the efforts to prevent and control anemia need to address preferentially most vulnerable age groups of children, under five years of age.

This review showed that the prevalence of anemia varied according to some predator factors from children and their families showing statistically significant variations for some factors and not for others. We have found that the magnitude of anemia does not vary with the gender of children. However, the educational status of children's families was a statistically significant predictor for the occurrence of anemia in children. Those children whose parents had no education were more likely to be anemic as compared to parents with primary educational status and above. This is congruent with a study done in Kenya 103 and Bangladesh 104. The reason could be explained that as the educational status of parents increases, so do their economic status and nutritional diversity. This results in an improved feeding practice and better health care of the children 105,106. Children from low household income families were more likely to be anemic as compared to those children from moderate and high-income families. This finding is concordant with the study conducted in Brazil 107 and in poor provinces of Qinghai and Ningxia from rural China 108. The reason could be because of the fact that parents of low household income may not secure balanced and nutrient-rich food stuffs and children's diet is usually monotonous^109^–^111^.

Moreover, the pooled results of five studies revealed that children from rural areas were 3.25 times more likely to be anemic than their urban counterparts. This finding is in harmony with what was reported by EDHS 2016 9. The reason could possibly be pertaining to inadequate information about healthy diet feeding, and other economic factors that could cause anemia in rural communities. Lastly, children infected with helminths are more likely to be anemic as compared to those non-infected ones. This is in trajectory with the studies conducted in Tanzania and Nigeria 112. The reason could be due to the fact that helminths infection causes loss of appetite, nutrition competition, feeding problem and red blood cell destruction in children. In addition, the parasites also result in impaired nutrient absorption by directly damaging the intestinal mucosal cells 113, 114.

## Strengths and limitations of the study

This study has a sort of strength in that it uses multiple databases not to miss any eligible study. Data extraction was also done reproducibly using a preset and pretested checklist so as to minimize errors that could affect the estimate. This systematic review and meta-analysis also included studies from different regions of the country, both published and unpublished articles. However, the study is not free from potential limitations, it is restricted to articles published in English language for example. In addition, since meta-analysis uses aggregated group data by its nature, other confounding factors which could affect anemia were not proscribed. This may have affected the estimate. Also, the articles included in this review are weak to establish causal relationship between the associated factors and the outcome for they are cross sectional by design. As a result, the results of this met-analysis is helpful if interpreted considering both inherent limitations of the original studies and the current meta-analysis.

## Conclusion

Over one third of children suffer from anemia and it is associated with low literacy, low socioeconomic status as well as rural residence of the families and helminthic infection of children. However, we did not find gender of the children as a statistically significant predictor of anemia among the children. Hence, anemia is a moderate public health problem in children in this study. The observed magnitude of anemia is likely to affect the intellectual potential and overall health of the children. Relevant community and school-based interventions should be strengthened to improve the problem. In addition, further longitudinal studies with long period follow-up are necessary so as to better explore more on the determinants of anemia in the study subjects in resource limited settings for successful interventions.

## Data Availability

All relevant data are within the paper and its Supporting Information files.

## Abbreviations

PRISMA: Preferred Reporting Items of Systematic Reviews and Meta-Analysis; EDHS: Ethiopian Demographic and Health Survey; JUSH: Jimma University Specialised Hospital; SNNPE: Southern Nations, Nationalities and peoples of Ethiopia; USA: United States of America; WHO: World Health Organization
